# Effect of Inhaling *Cymbopogon martinii* Essential Oil and Geraniol on Serum Biochemistry Parameters and Oxidative Stress in Rats

**DOI:** 10.1155/2014/493183

**Published:** 2014-12-09

**Authors:** Bruna Fernanda Murbach Teles Andrade, Camila Pereira Braga, Klinsmann Carolo dos Santos, Lidiane Nunes Barbosa, Vera Lúcia Mores Rall, José Maurício Sforcin, Ana Angélica Henrique Fernandes, Ary Fernandes Júnior

**Affiliations:** ^1^Department of Microbiology and Immunology, Institute of Biosciences, UNESP, 18618-970 Botucatu, SP, Brazil; ^2^Department of Chemistry and Biochemistry, Institute of Biosciences, UNESP, 18618-970 Botucatu, SP, Brazil

## Abstract

The effects of the inhalation of *Cymbopogon martinii* essential oil (EO) and geraniol on Wistar rats were evaluated for biochemical parameters and hepatic oxidative stress. Wistar rats were divided into three groups (*n* = 8): G1 was control group, treated with saline solution; G2 received geraniol; and G3 received *C. martinii* EO by inhalation during 30 days. No significant differences were observed in glycemia and triacylglycerol levels; G2 and G3 decreased (*P* < 0.05) total cholesterol level. There were no differences in serum protein, urea, aspartate aminotransferase activity, and total hepatic protein. Creatinine levels increased in G2 but decreased in G3. Alanine aminotransferase activity and lipid hydroperoxide were higher in G2 than in G3. Catalase and superoxide dismutase activities were higher in G3. *C. martinii* EO and geraniol increased glutathione peroxidase. Oxidative stress caused by geraniol may have triggered some degree of hepatic toxicity, as verified by the increase in serum creatinine and alanine aminotransferase. Therefore, the beneficial effects of EO on oxidative stress can prevent the toxicity in the liver. This proves possible interactions between geraniol and numerous chemical compounds present in *C. martinii* EO.

## 1. Introduction

Plants synthesize around 200,000 secondary metabolites or specialized phytochemicals, of which essential oils (EOs) constitute an important group [1]. These compounds can be extracted from plant tissues (e.g., stem, leaves, flowers, and roots) by several procedures (e.g., hydrodistillation and steam distillation) [2]. These compounds are mostly terpenes, which are commonly used in pharmaceutical industries and have therapeutic benefits and promote welfare, especially when used in aromatherapy procedures [3].

*Cymbopogon martinii* (Roxb.), Watson, popularly known as palmarosa, exhibits beneficial effects on several central nervous system pathologies, mainly neuralgia, epileptic, and anorexia [4]. There are a few reports on its effects; still* C. martinii* has attracted many researchers' attention due to its antimicrobial, antigenotoxic, and antioxidant activities [5–8]. Countries such as India, Brazil and Madagascar have the practice to produce EOs from this plant.

Geraniol, the major constituent of* C. martinii* EO, is an acyclic monoterpenoid that is abundant in many plants [9]. It may represent a new class of therapeutic agents against pancreatic [10] and colon cancers [11] and has several biological properties, including antimicrobial, antioxidant and anti-inflammatory activities [12]. Geraniol is also an important constituent of ginger, lemon, lime, lavender, nutmeg, orange and rose EOs [13]. Also, it is used as a flavoring agent and was determined to be safe at the current levels of intake by the Joint Expert Committee on Food Additives of Food and Agriculture Organization—FAO/World Health Organization—WHO [14].

Aromatherapy is a traditional treatment that uses EOs. Its effects begin when the aromatic molecule passes through the nasal cavity and adheres to the olfactory epithelium, causing nerve stimulation directly to the hippocampus and limbic amygdaloidal body. This consequently triggers stimuli that control the autonomic nervous system and internal secretory control by changing a number of vital reactions [15]. The inhalation of aromatic compounds present in EOs is the reason for the name “aromatherapy” and this therapy may have sedating or stimulating effects on the individual [16].

Reports in the literature describe the benefits of using EOs in aromatherapy on the wellbeing of individuals, including improvements in mood, stress, anxiety, depression, and chronic pain, and promote so therapeutic, psychological, and physiological effects [17]. The inhalation of EOs elevated blood pressure and renal sympathetic activity, which enforces the idea that these components act in the central nervous system and pass through the blood-brain barrier [18].

Volatile organic compounds are highly lipophilic and may easily cross the blood-brain barrier and easily exert their neuropharmacological and toxicological effects. While studies on the toxic effects of these compounds are relatively easy to perform, the central effects induced by the perception of odor (e.g., in aromatherapy) are inherently complex. This is why the toxicological studies performed using volatile compounds are much more advanced [19].

Many studies have been conducted* in vitro* with the purpose of verifying the biological properties of EOs [2, 17, 20]; usually they are performed using* in vitro* assays. On the other hand, when the tests are performed* in vivo*, the products are usually administered in their liquid forms (e.g., by gavage or intraperitoneal), with few studies in the volatile state (i.e., by inhalation) [21]. Furthermore, EOs are used as flavoring agents in food products [14] and are also used in dermatology and in the fragrance and cosmetics industries. Specifically, geraniol is extensively used in the manufacture of both household and cosmetics products [12].

Since people are often use EOs, it is important to evaluate the possible hepatotoxic effects of these oils. Liver is the main detoxification organ; the catabolism of both endogenous and exogenous compounds takes place in the liver. As a result it is exposure to toxic agents which can cause drug-induced hepatic dysfunction. Therefore, studies on serum activity of alanine aminotransferase (ALT) and aspartate aminotransferase (AST), which are biomarkers of liver damage, are important. The serum activity of ALT and AST is frequently used in clinical settings for diagnostic hepatic toxicity [22].

Numerous chronic degenerative diseases are associated with oxidative stress, which occurs when there is excess formation of reactive oxygen species (e.g., superoxide, hydroxyl, and hydrogen peroxide) and insufficient defense by the antioxidant system (enzymatic and nonenzymatic). This imbalance between pro- and antioxidants may cause cell injury and death, which consequently lead to tissue dysfunction [23, 24]. It is well established that oxidative stress plays a fundamental role in the pathogenesis of hepatic disease, especially nonalcoholic steatohepatitis [25]. In addition, during hepatic catabolism of xenobiotics, excessive production of reactive oxygen species (ROS) occurs [26].

Our aim was to investigate the effect of inhalation of the* C. martinii* EO and geraniol on serum biochemical parameters, biomarkers of hepatotoxicity, and oxidative stress in hepatic tissue.

## 2. Materials and Methods

### 2.1. *Cymbopogon martinii* EO and Geraniol

*C. martinii* EO was supplied by the company* By Samia Aromaterapia* (São Paulo, SP) and showed the following chemical composition: geraniol (57.5%), geranyl acetate (13.6%), linalool (1.7%), *β*-caryophyllene (1.1%), and ocimene (0.3%) found by gas chromatography-mass spectrometer (GC-MS). The geraniol with 98% of purity was purchased from Sigma Aldrich (St. Louis, MO, USA).

### 2.2. Animals and Experimental Procedure

The experimental procedure was approved by the Ethical Committee from Institute of Biological Science, São Paulo State University, Botucatu, Brazil, and the animals experiments were carried out in accordance with the principles and guidelines of the Canadian Council on Animal Care as outlined in the Guide to the Care and Use of Experimental Animals.

Male Wistar rats (290–310 g) were reared in polypropylene cages maintained in a controlled environment (temperature 22 ± 3°C; 50–55% humidity; and a 12-hour light : dark cycle), with free access to water and food (Purina Ltd., Campinas, SP, Brazil).

The rats were randomly distributed into three groups (*n* = 8). The rats in the control group (G1) received saline solution by inhalation (saline = 0.9% g/v). The G2 group received geraniol by inhalation and the G3 group received* C. martinii* EO by inhalation.

The rats from all groups were placed individually into chambers (180 mm × 300 mm × 290 mm) adapted from de Almeida et al. [27] and submitted to inhalation of geraniol (8.36 mg geraniol/L of air, which corresponds to 136.2 *μ*L of geraniol/perspex box 14.5 L of air) and* C. martinii* EO (13.73 mg of* C. martinii* EO/L of air, which corresponds to 227 *μ*L of* C. martinii* EO/perspex box 14.5 L of air) for 10 minutes every 48 hours for 30 days. The geraniol concentration was calculated from the amount of geraniol found in the* C. martinii* EO.

Food and water consumption were measured daily at the same time and body weights were determined once a week.

### 2.3. Biochemical Measurements and Oxidative Stress

After 30 days, the animals were fasted overnight (12–14 h) and euthanized by cervical decapitation under anesthesia (solution containing 10% ketamine chloride and 2% xylazine chloride with a dose of 0.1 mL/100 g body weight). Blood was collected and the serum was obtained by centrifugation at 6000 rpm for 15 minutes. Serum glucose was determined using an enzymatic colorimetric method after incubation with glucose oxidase/peroxidase. The total amount of protein was estimated using the biuret reagent and the total cholesterol concentration was determined using the cholesterol esterase/oxidase enzymatic procedure. Triacylglycerols levels were measured by enzymatic hydrolysis and the final formation of quinoneimine, which is proportional to the concentration of triacylglycerols present in the sample. Serum urea was determined by addition of urease and phenol-hypochloride, which leads to the formation of an indophenol-blue complex. The serum creatinine levels were estimated using a reaction with picric acid in alkaline buffer to form a yellow-orange complex, whose color intensity is proportional to the creatinine concentration in the sample. ALT and AST activities were determined by using pyruvate and oxaloacetate as substrates, wherein NADH is converted into NAD+ proportional to the activities of these enzymes. Hepatic samples (200 mg) were removed and homogenized in 0.1 M phosphate buffer, pH 7.4, using a Teflon-glass Potter-Elvehjem homogenizer. The homogenate was centrifuged (10,000 g for 15 minutes) and the supernatant was used to determine the concentration of hepatic lipid hydroperoxide (LH) and activities of antioxidant enzymes. Lipid hydroperoxide activity was determined by the oxidation of Fe^+2^ in the presence of a reactive mixture containing methanol, xylenol orange, sulfuric acid, and butylated hydroxytoluene. Catalase activity was assayed using phosphate buffer containing hydrogen peroxide. The activity of glutathione peroxidase (GSH-Px) was determined in the presence of phosphate buffer, NADPH_2_, reduced glutathione, and glutathione reductase. Superoxide dismutase (SOD) activity was assayed according to the method by measuring the rate of reduction of nitroblue-tetrazole (NBT) in the presence of free radicals generated by hydroxylamine.

### 2.4. Statistical Analysis

Results are expressed as the mean ± SD. The statistical significance between the groups was assessed using one-way analysis of variance (ANOVA) with Tukey's test to compare the means of the experimental group. The probability with *P* ≤ 0.05 was considered significant.

## 3. Results

**Figure 1:**
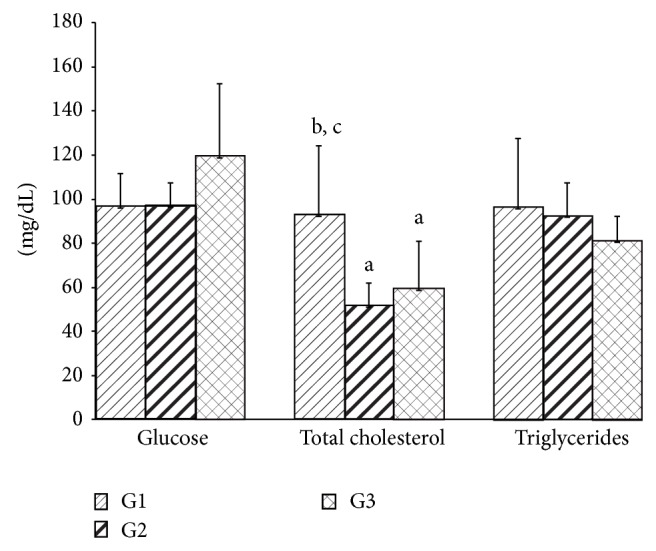
Serum glucose, total cholesterol, and triglycerides levels after 30 days for all experimental groups. Values are given as the mean ± SD for each group of eight animals. ^a^Significantly different from G1; *P* ≤ 0.05; ^b^significantly different from G2; *P* ≤ 0.05; and ^c^significantly different from G3; *P* ≤ 0.05. G1: untreated control; G2: treated with geraniol; and G3: treated with essential oil.

**Figure 2:**
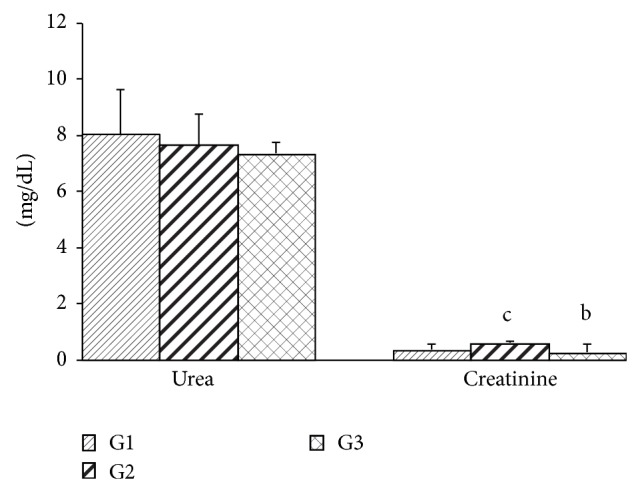
Serum urea and creatinine levels after 30 days for all experimental groups. Values are given as the mean ± SD for groups of eight animals each. ^a^Significantly different from G1; *P* ≤ 0.05; ^b^significantly different from G2; *P* ≤ 0.05; ^c^significantly different from G3; *P* ≤ 0.05. G1: untreated control; G2: treated with geraniol; G3: treated with essential oil.

Inhalation of geraniol (G2) and of* C. martinii* EO (G3) had no effects on final body weight, body weight gain, and food intake of the rats (Table 1). No alteration in total hepatic protein was observed. While no significant differences were observed in the glycemia and triacylglycerol levels, geraniol (G2) and* C. martinii* EO (G3) decreased (*P* ≤ 0.05) total cholesterol levels when compared with the control group G1 (Figure 1). There were no significant differences in serum urea levels between the groups. Creatinine levels increased in the presence of geraniol but decreased in the presence of* C. martinii* EO (G3; Figure 2).

**Figure 3:**
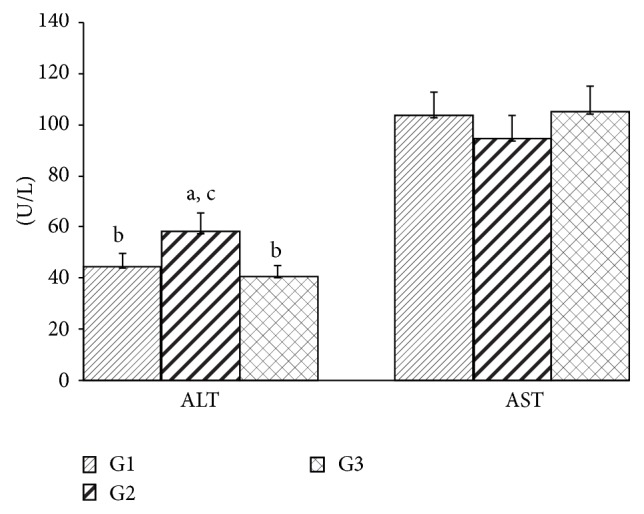
Serum activity of ALT and AST after 30 days for all experimental groups. Values are given as the mean ± SD for each group of eight animals. ^a^Significantly different from G1; *P* ≤ 0.05; ^b^significantly different from G2; *P* ≤ 0.05; and ^c^significantly different from G3; *P* ≤ 0.05. G1: untreated control; G2: treated with geraniol; and G3: treated with essential oil.

**Figure 4:**
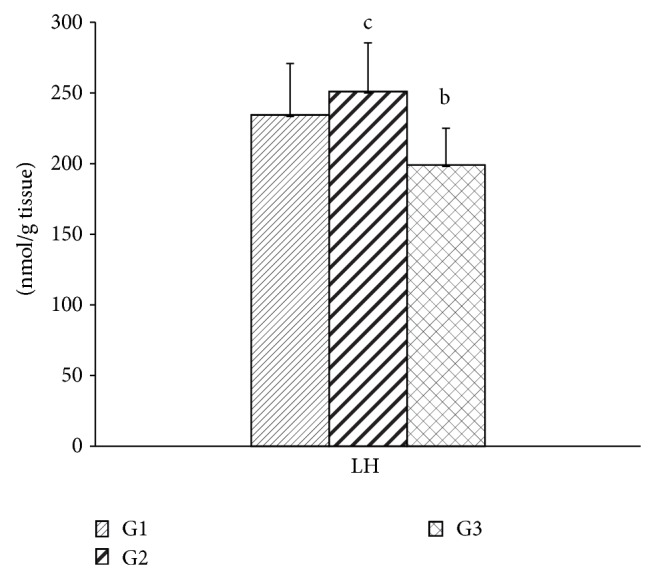
Hepatic lipid hydroperoxide levels after 30 days for all experimental groups. Values are given as the mean ± SD for each group of eight animals. ^a^Significantly different from G1; *P* ≤ 0.05; ^b^significantly different from G2; *P* ≤ 0.05; and ^c^significantly different from G3; *P* ≤ 0.05. G1: untreated control; G2: treated with geraniol; and G3: treated with essential oil.

**Figure 5:**
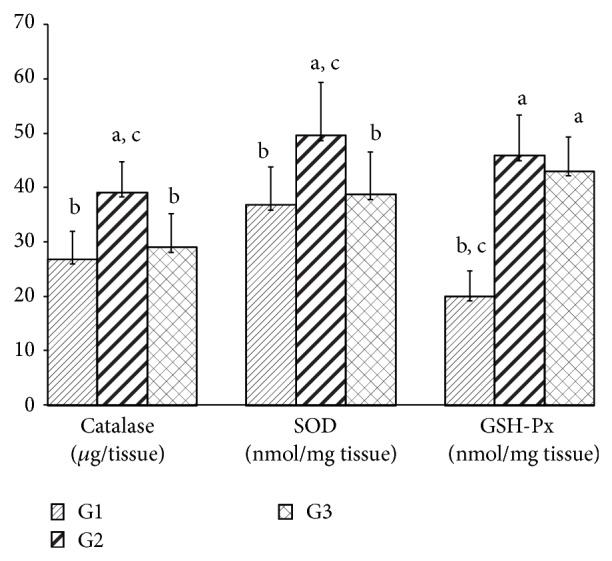
Hepatic activities of catalase, SOD, and GSH-Px after 30 days for all experimental groups. Values are given as the mean ± SD for each group of eight animals. ^a^Significantly different from G1; *P* ≤ 0.05; ^b^significantly different from G2; *P* ≤ 0.05; and ^c^significantly different from G3; *P* ≤ 0.05. G1: untreated control; G2: treated with geraniol; and G3: treated with essential oil.

ALT activity was higher in the group exposed to geraniol when compared to the other groups, which did not differ from each other. No change was found in the AST activity between the groups (Figure 3). LH was higher in the G2 group than in the G3 group (Figure 4). Catalase and SOD activities were higher in the G3 group when compared to the other groups. Both geraniol and* C. martinii* EO increased GSH-Px when compared to the control rats (Figure 5).

## 4. Discussion

EOs are widely used in aromatherapy procedures and there is interest in reports on the hepatic toxicity of these natural products. However, few studies have explored the effects of EOs on an organism when administered by inhalation. Since EOs have been extensively used in aromatherapy due to their therapeutic properties and also used in food products, in dermatology, and in the fragrance and cosmetic industries [14], there is interest in investigating their hepatic toxicity, as well as dyslipidemia.

*C. martinii* EO reduced the final water intake of the rats, but without altering other parameters that we studied. No significant changes were observed in final body weight, body weight gain, final food intake, or total serum protein level, indicating no dehydration and no deficiencies of nutritionally important compounds in animals in the experimental groups. These results disagree with others studies, which showed that the use of different EOs, for example,* Citrus aurantifolia* EO, decreases food intake and, consequently, weight gain [28, 29].

There were no changes in serum glucose levels between the groups, indicating maintenance of glycemic homeostasis in these animals. However, other experimental studies have demonstrated that* C. martinii* extracts exhibit antihyperglycemic activities through inhibition of *α*-glucosidase under diabetic conditions [30]. Assays with* C. citratus* aqueous extract (500 mg/kg/day; via oral) showed that the mechanism by which the extract induced hypoglycemia could be attributed to increased insulin synthesis and secretion or increased peripheral glucose utilization [31].

The inhalation of both geraniol (G2) and* C. martinii* EO (G3) reduced total cholesterol, but no changes in the serum triacylglycerol concentration were observed. Our results are in agreement with the result of Adeneye and Agbaje [31] and Burke et al. [10], who observed hypocholesterolemic effects using an aqueous extract of* Cymbopogon citratus* by oral administration. This reduction caused by EO can possibly be attributed to inhibition of 3-hydroxy-3-methylglutaryl CoA reductase, a key enzyme that regulates hepatic cholesterol synthesis [32, 33], or by reduction in the expression of these enzymes [34]. Yu et al. [35] demonstrated that geraniol inhibited the formation of mevalonate, a metabolic intermediate in the biosynthesis of cholesterol, in hepatomas. On the other hand, the administration of the highest EO dose (100 mg/kg) from* Cymbopogon* resulted in no change in total serum cholesterol [36].

Our results are also in agreement with Costa et al. [36] about total serum cholesterol; they reported that the biochemical parameters did not change after treatment with* Cymbopogon* but showed that there was a significant reduction in it (*F*(4,27) = 3.061; *P* ≤ 0.05) after the administration of the highest EO dose (100 mg/kg) by gavage over a period of 21 days.

Since urea is formed in the liver and excreted by kidneys, estimation of this nitrogenous compound in the bloodstream is important to estimate both hepatic and renal functions. Animals treated with geraniol and EO did not show altered serum urea levels, suggesting a normal degree of protein catabolism, which was confirmed by a normal concentration of hepatic protein. Although there was no alteration in the levels of serum urea in the G2 group, we cannot exclude the involvement of possible changes in the glomerular filtration rate in these animals. In clinical practice, serum creatinine, a biomarker for renal failure, is used as an indicator of renal function [37]. Curiously, serum creatinine levels were higher in the G2 group and lower in the G3 group, suggesting a decrease in renal excretion and some degree of renal insufficiency or early stages of kidney dysfunction when the major compound of* C. martinii* EO was administrated alone. The animals treated with both products had a tendency to have decreased levels of serum urea in our study. Serum creatinine was higher in G2, which may indicate lower renal excretion since the creatinine production was relatively constant [38].

Plasma membrane damage from some cells types, such as hepatic cells, is accompanied by release of cytosolic enzymes into bloodstream, a phenomenon that always occurs under several pathophysiological conditions [39]. The aminotransferases ALT and AST are used for diagnosis of hepatic injury after toxic agents exposure [40]. There was a significant increase in serum ALT activity in animals that inhaled geraniol (Figure 3). Since serum enzymatic activity of ALT is often used as a biomarker of hepatic toxicity, we can assume that there was some injury in hepatic tissue induced by geraniol after 30 days.

ROS, such as superoxide anion (O_2_^−^), hydroxyl radicals (OH^−^), and hydrogen peroxide (H_2_O_2_), are formed through mitochondrial respiration during normal cellular metabolism. However, the cells have an enzymatic antioxidant defense system against ROS, but, under certain pathological conditions the excess formation of ROS results in suppression of antioxidant enzymes, the increase of ROS can occur in this way leading to oxidative stress [41].

Lipid peroxidation is an important toxic event because involves the removal of hydrogen from fatty acid chains mediated by ROS [42, 43] this way can lead to cell death and tissue damage.

The endogenous antioxidant enzyme includes superoxide dismutase that catalyzes the dismutation of superoxide radicals [23]. Glutathione peroxidase catalyzes the reduction of hydrogen peroxidase to water through the oxidation of reduced glutathione. Catalase also participates in this conversion [44].

Significantly high LH was observed in rats exposed to geraniol (G2), while the beneficial effect of* C. martinii* EO was evidenced by the reduced LH in these animals. The reductions observed in G3 can be attributed to a synergistic mechanism: a concomitant antioxidant action between other compounds, for example, linalool and *β*-caryophyllene, present in the* C. martinii* EO that showed antioxidant activity in other researches [45, 46]. Since free radical scavenger ability depends on the number of hydroxyl radicals in the molecule [43], the inhalation of the total* C. martinii* EO contributed to the reduction in the formation of ROS.

Rats exposed to geraniol (G2) had higher catalase, SOD, and GSH-Px activities, indicating that antioxidant enzyme activities were not sufficient to inhibit the ROS action and, consequently, the lipoperoxide generation in liver of these animals.

According to Koek et al. [47] the activity of antioxidants enzymes is increased early in nonalcoholic steatohepatitis but tends to decrease with progression of pathogenesis. The activity of the SOD and catalase did not change in the G3 group, while GSH-Px increased in these animals, which showed lower values for LH. Buch et al. [4] observed increases in both SOD and catalase in the brains of rats treated with* C. martini* EO. Terpenoids, which are important components of EOs, lowered malondialdehyde levels and improved SOD activity in gastric mucosa [48]. Experimental data have shown that terpenoids, which are main components of EOs, are responsible for their antioxidant action [49, 50].

Since lipid hydroperoxide has been widely studied as marker of lipoperoxidation [51], a process that involves removal of hydrogen from fatty acids side chains by ROS, the result is referring to the mixture of compounds present in* C. martinii* EO (G3) that was effective in controlling oxidative stress and, therefore, lipoperoxidation by reducing the concentration of LH through a mechanism independent of the endogenous antioxidant enzymatic system.

In another study, geraniol reduced lipid peroxidation and inhibited the release of NO, indicating its possible antioxidant potential in inflammatory lung diseases, in which oxidative stress plays key role in these pathogenesis [14]. Moreover, the possible synergism between the compounds present in EOs can influence biological responses [52]. This can explain the results obtained for the G3 group. Thus, the effects we observed could be attributed to a constituent in a smaller proportion or synergism between compounds that are present in the oil [53]. For biological purposes, it is more informative to study the whole oil than some of its components because the concept of synergism appears to be more significant in the research on natural products [2].

## 5. Conclusion

In conclusion,* C. martinii* EO and geraniol maintained the glycemia, triacylglycerol protein, and urea levels but decreased cholesterol levels in Wistar rats. The oxidative stress caused by geraniol alone appears to trigger, to some degree, hepatic toxicity, as can be verified by the increase of serum creatinine and ALT. These results suggest that beneficial actions of* C. martinii* EO on oxidative stress can prevent the toxicity in liver. This proves the possible interactions between geraniol and numerous chemical compounds present in* C. martinii* EO.

## Figures and Tables

**Table 1:** General characteristics and serum protein levels after 30 days for all experimental groups.

Parameters	Groups		
	G1	G2	G3
Final body weight g	348.57 ± 43.65	328.40 ± 29.82	320.98 ± 39.90
Body weight gain g	37.47 ± 9.25	36.86 ± 8.22	38.53 ± 17.06
Final food consumption g/day	20.30 ± 4.40	19.25 ± 4.14	18.52 ± 3.78
Final water consumption mL/day	248.75 ± 21.84^c^	248.75 ± 16.20^c^	211.88 ± 10.67^a,b^
Serum protein g/dL	5,13 ± 0,83	5,39 ± 1,39	5,95 ± 1,13

Values are given as the mean ± SD for each group of eight animals. ^a^Significantly different from G1; *P* ≤ 0.05; ^b^significantly different from G2; *P* ≤ 0.05; and ^c^significantly different from G3; *P* ≤ 0.05. G1: untreated control; G2: treated with geraniol; and G3: treated with essential oil.

## Abbreviations

*C. martinii*: * Cymbopogon martinii*
EO: Essential oil
EOs: Essential oils
ALT: Alanine aminotransferase
AST: Aspartate aminotransferase
ROS: Reactive oxygen species
LH: Lipid hydroperoxide
GSH-Px: Glutathione peroxidase
SOD: Superoxide dismutase
NBT: Nitroblue-tetrazole.
